# An artificial mucus-integrated *in vitro* model enables stable live probiotic–host co-culture and recapitulates dynamic hBD-2-mediated antimicrobial feedback

**DOI:** 10.1080/29933935.2026.2622881

**Published:** 2026-01-31

**Authors:** Wenxin Cao, Yumiko Watanabe-Yasuoka, Toshihiro Sashihara, Masaki Nishikawa, Yasuyuki Sakai

**Affiliations:** a Department of Chemical System Engineering, Graduate School of Engineering, University of Tokyo, Tokyo, Japan; b Wellness Science Labs, Meiji Holdings Co., Ltd., Tokyo, Japan

**Keywords:** *In vitro* intestinal model, host-microbe interaction, artificial mucus, human β-defensin-2, probiotic screening

## Abstract

*In vitro* models for host–microbe interaction often have limited physiological relevance due to the absence of a protective mucus layer, reliance on non-viable bacteria, and short co-culture durations. Here, we present the first scalable and biocompatible *in vitro* model integrating an artificial mucus barrier that enables stable 48-hour co-culture of live *Lactiplantibacillus plantarum* OLL2712 with Caco-2 cells at exposure ratios up to 1,000. This model maintained epithelial viability, supported bacterial proliferation, and enabled dynamic host responses. Notably, during co-culture, while hBD-2 gene expression was observed under both with-mucus and without-mucus conditions, protein secretion occurred only in the presence of mucus, reaching approximately a 100-fold higher level than previously reported. This finding underscores the essential role of the mucus barrier in preserving epithelial function and maintaining the downstream host response. The model recapitulates a complete host-microbe feedback loop involving microbial stimulation, antimicrobial peptide (hBD-2) secretion, and subsequent bacterial suppression, thereby linking epithelial defense activation to microbial regulation. It provides a reproducible, physiologically relevant, and animal-free platform for probiotic screening and mechanistic studies of mucus-associated intestinal disorders such as inflammatory bowel disease.

## Graphical abstract

**Figure:**
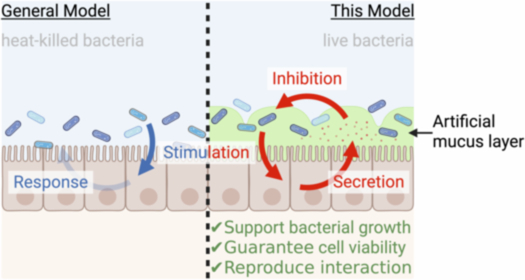

## 1 Introduction

The human gastrointestinal (GI) tract hosts a highly diverse and dynamic microbial ecosystem that plays essential roles in nutrient absorption, immune regulation, and maintaining mucosal homeostasis.^1^^,^^2^ One of the key areas for these host–microbe interactions is the small intestine, where a thin, specialized mucus layer directly interfaces with both luminal contents and epithelial surfaces. This mucosal environment is optimized for selective permeability and efficient digestion but it depends on a carefully regulated immune balance to prevent excessive inflammation from pathogenic substances.^3^ Disruptions in this balance, caused by microbial imbalance, mucosal barrier degradation, or immune dysregulation, have increasingly been linked to conditions such as inflammatory bowel disease (IBD), metabolic syndromes, and colorectal cancer.^4^^,^^5^

The intestinal mucus layer, mainly composed of secreted mucins (~0.2 to 5.0% w/v), water (~95% w/w), globular proteins (~0.5% w/v), salts (~0.5 to 1.0% w/w), and lipids (1–2% w/w),^6^ acts as the first line of defense by physically separating the epithelium from the microbiota community in the lumen.^7^ Beyond its structural role, this layer also actively influences microbial adhesion, controls access to epithelial surfaces, and aids in antimicrobial defense.^8^ Importantly, clinical observations in IBD patients consistently show changes in mucus layer integrity, including thinning of the mucus barrier, increased epithelial permeability, and mucus presence in fecal discharge, indicating a causal link between mucus degradation and disease progression.^9^

Despite growing recognition of mucus-related dysbiosis in disease progression, current tools for investigating these mechanisms are still limited. *In vivo* models lack real-time control and accessibility, while many *in vitro* systems cannot incorporate live bacterial dynamics because of the risks of cytotoxicity. Instead, they often rely on microbial components like lipopolysaccharide (LPS) or flagellin,^10^^,^^11^ which fail to capture the physiological dynamics of microbial metabolism, proliferation, and immune modulation that happen during live host–microbe interactions. As a result, the real-time interactions between epithelial defense responses and bacterial behavior remain poorly understood.^12^ Attempts at long-term live co-culture studies usually cause rapid epithelial damage *in vitro* because of the absence of a protective barrier.

Therefore, there is a critical need for a controllable and scalable *in vitro* co-culture model that supports sustained host–microbe interactions with a functional mucus barrier under well-defined conditions. To address these challenges, conventional approaches typically rely on three main strategies: harvesting native mucus from animal tissues, inducing mucus secretion through goblet cell co-culture, or using commercial mucin products. Native mucus is a biologically complex substance, but its composition varies inconsistently across batches and species, resulting in low reproducibility and physiological relevance.^13^ Cell models such as Caco-2/HT29-MTX/Raji B triple co-cultures that introduce goblet-like cells or iPSC-derived intestinal epithelium with mucus-secreting capacity are limited by low and inconsistent secretion volumes and thickness.^14^ Commercially available, purified mucin products are widely used due to their simplicity and accessibility, offering a more standardized option for mucus modeling. However, they lack the biological complexity of native mucus due to the irreversible disruption of the mucin network structure during the purification process, which significantly limits their functional relevance for comprehensive host-microbe interaction studies.^15^^,^^16^

To overcome these limitations, we aimed to develop a practical *in vitro* model of the small intestine by incorporating an artificial mucus layer onto Caco-2 epithelial monolayers. Live *Lactiplantibacillus plantarum* OLL2712 was introduced as a model probiotic for microbial colonization. We systematically assessed the model's performance over a 48-hour co-culture period using a combination of assays for epithelial viability and function, bacterial growth analysis, and the expression and secretion of human β-defensin-2 (hBD-2), an inducible antimicrobial peptide secreted by intestinal epithelial cells in response to microbial stimulation, serving as a key marker for host-microbe interaction in this study. The preparation of the artificial mucus layer was adapted and modified from the hydrogel formulation described by Boegh et al.^17^ Building upon previous studies that primarily focused on short-term structural or rheological evaluation, we conducted a comprehensive biocompatibility and functional validation over a 48-hour co-culture period, significantly extending the duration and physiological relevance of our study beyond previous research.^18^ More importantly, to our knowledge, we present a novel *in vitro* co-culture system that is the first to support 48-hour live interactions between intestinal epithelial cells and probiotic bacteria in the presence of a physiologically relevant mucus layer, systematically investigating dynamic host-microbe crosstalk.

Our results demonstrated that the artificial mucus supported active bacterial proliferation while preserving epithelial viability and barrier function, avoiding the cytotoxic effects commonly seen in long-term co-cultures. Notably, epithelial secretion of hBD-2 was only detectable in the presence of mucus, highlighting the system's capacity to support functional host responses. Furthermore, the co-culture exhibited a feedback mechanism, where excessive bacterial growth was suppressed when epithelial cells were present. Together, based on previous studies focused mainly on short-term structural evaluation, we developed a mucus-integrated model that supports stable 48-hour co-culture between live bacteria and epithelial cells, thereby extending the physiological relevance of *in vitro* host-microbe interaction studies. Its scalability, modularity, and animal-free design make it an ideal platform for mechanistic microbiota research, drug screening, and disease modeling—particularly for IBD, where mucus thinning and microbial dysbiosis are critical pathological features.

## 2 Materials and methods

### 2.1 Preparation of artificial mucus

The artificial mucus was adapted from the biosimilar mucus proposed by Boegh et al.^17^ with key modifications to suit long-term co-culture and static *in vitro* conditions. The lipid mixture mentioned in earlier research was excluded to minimize cellular toxicity and accommodate the static co-culture model setup in this study, since the lipid components, including linoleic acid, cholesterol, and phosphatidylcholine, were introduced initially to mimic the hydrophobic nature and viscoelastic properties of native mucus, particularly under shear stress conditions mimicking peristalsis. However, in our static co-culture model, such mechanical resilience was not required, and preliminary biocompatibility tests demonstrated increased cytotoxicity associated with lipid addition during prolonged incubation.

The final composition and concentrations of the artificial mucus were thus optimized through serial biocompatibility tests on the Caco-2 monolayer over a 48-hour period. To prepare a 40 mL artificial mucus sample, 3 g lyophilized mucin from porcine stomach (Sigma Aldrich) was first autoclaved at 121 °C, 15 psi for 15 minutes, then dissolved in 22.5 mL sterilized isotonic buffer of 10 mM 4-(2-hydroxyethyl)-1-piperazineethanesulfonic acid (HEPES) with 1.3 mM CaCl_2_, 1.0 mM MgSO_4_, and 40 mM NaCl in ultrapure water (Milli-Q), under magnetic stirring for about 5 hours to ensure complete dissolution. 0.6 g polyacrylic acid (PAA, Carbopol® 974P NF from Lubrizol) was dissolved in 20 mL of the same buffer with magnetic stirring under UV light for 1 hour at room temperature until fully dissolved. 1.86 g bovine serum albumin (BSA, Sigma Aldrich) was dissolved in 6 mL isotonic buffer and sterilized through a 0.45 μm filter. After each component was thoroughly mixed and sterilized, 15 mL PAA was slowly added to the 15 mL mucin solution with stirring. 1 M Sodium hydroxide (NaOH) was then added to raise the pH to approximately 7. 4 mL BSA was added last with gentle stirring. Once thoroughly mixed, a certain amount of NaOH was added again to adjust the final pH to 7.4, resulting in the mucus mixture. The final artificial mucus contains 5% (w/v) mucin, 0.9% (w/v) PAA, and 3.1% (w/v) BSA. The mixture was stored overnight at 4 °C before use in subsequent studies.

### 2.2 Biocompatibility analysis

Glucose and L-lactate concentrations in the culture supernatants were measured using a biochemical analyzer (Model: BF-48AS/T, No. AS48034, Ohji Keisoku Kikai Co., Ltd., Japan). Before sample analysis, calibration was performed with 200 μL of Milli-Q water (blank) and a series of glucose and L-lactate standard solutions at known concentrations. For each sample, 100 μL of culture supernatant was loaded into the instrument following the manufacturer's instructions. Measurements were automatically processed by the instrument, and the concentration values were reported directly in mmol/mL. Glucose consumption and L-lactate production were calculated accordingly based on the standard values measured in the cell culture medium.

Cell cytotoxicity was measured using a colorimetric lactate dehydrogenase (LDH) assay kit (Dojindo Laboratories). Basolateral medium was collected after 48 hours of exposure, and LDH levels were determined following the manufacturer's instructions. Absorbance was read at 490 nm using a Bio-Rad iMark™ Microplate Absorbance Reader, and results were normalized to positive (lysis buffer-treated) and negative (untreated) controls. All measurements were conducted in transparent 96-well plates with triplicate technical replicates for each condition.

Barrier integrity of the Caco-2 epithelial monolayer was evaluated using a 4 kDa FITC-dextran permeability assay. After 48 hours of mucus exposure, 1 mg/mL FITC-labeled dextran (4 kDa, Sigma-Aldrich) in complete culture medium was added to the apical chamber of the Transwell insert. Following a 4-hour incubation at 37 °C, 100 μL of medium was collected from the basal compartment for analysis. Fluorescence intensity was measured with a microplate fluorometer (Model No. 4201852), using a standard 490/534 nm fluorometry protocol. Permeability was calculated by interpolating fluorescence values onto a standard curve generated from serial dilutions of FITC-dextran standards. All measurements were performed in black 96-well plates with triplicate technical replicates for each condition.

### 2.3 Co-culture of epithelial cells and intestinal bacteria

Caco-2 cells (RCB0988, RIKEN BRC cell bank) were maintained in high-glucose Dulbecco’s Modified Eagle Medium (DMEM) supplemented with 10% fetal bovine serum (FBS), 1% nonessential amino acid (NEAA), and 1% Penicillin-Streptomycin-Amphotericin B solution (PSA), in a humidified incubator at 37 °C with 5% CO₂. For experiments, Caco-2 cells were seeded at a density of 1 × 10⁵ cells/cm² onto 24-well cell culture inserts with a surface area of 0.33 cm² (THINCERT®, Geriner Bio-One) and cultured for 14 d to allow monolayer formation and differentiation. Prior to bacterial inoculation, the culture medium was replaced with PSA-free medium to permit live co-culture.

*Lactiplantibacillus plantarum* OLL2712, provided by Meiji Co., Ltd. (Tokyo, Japan), were cultured in de Man, Rogosa, and Sharpe (MRS) broth (Sigma Aldrich) at 37 °C under anaerobic conditions using AnaeroPack® (Mitsubishi Gas Chemical Co., Inc.) for at least 16 hours prior to use. The bacterial cells were collected by centrifugation (8,000 × g, 5 minutes), washed twice with sterile phosphate-buffered saline (PBS), and resuspended in PSA-free culture medium to achieve concentrations of 10^6^, 10^7^, 10^8^ CFU/mL. Viable counts were verified by serial dilution and colony-forming unit (CFU) enumeration on MRS agar after 24–48 hours of incubation at 37 °C.

For the co-culture experiments, bacterial suspensions at the specified concentrations were added aseptically to the apical chamber of the Transwell inserts containing differentiated Caco-2 monolayers, with or without the presence of the artificial mucus layer. The co-culture was maintained for 48 hours at 37 °C. After incubation, various assays were performed to assess epithelial viability, barrier integrity, and host–microbe interactions, and the results were compared across different culture conditions.

### 2.4 hBD-2 regulation qualification and quantification

Total RNA was extracted from Caco-2 monolayers after co-culture using TRIzol™ Reagent (Invitrogen) following the manufacturer's instructions. RNA quantity and purity were assessed using a NanoDrop spectrophotometer (Thermo Fisher Scientific). Complementary DNA (cDNA) was synthesized from the extracted RNA using the PrimeScript™ RT Reagent Kit (Takara Bio) following the manufacturer's instructions, using 800 ng of input RNA per reaction.

Quantitative real-time PCR (RT-qPCR) was conducted using the KOD SYBR® qPCR Mix (Toyobo) on a StepOnePlus Real-Time PCR System (Applied Biosystems). hBD-2 expression was determined with the following primers: forward 5′-GGTATAGGCGATCCTGTTACCTGC-3′ and reverse 5′-TCATGGCTTTTTGCAGCATTTTGTTC-3′. Relative gene expression levels were calculated using the 2^-ΔΔCt method with β2-Microglobulin (B2M) as the internal control.

To evaluate hBD-2 secretion at the protein level, apical supernatants were collected and analyzed using a human β-defensin-2 ELISA kit (Phoenix Pharmaceuticals), following the manufacturer's instructions. Protein levels were compared with mRNA expression to assess host epithelial responses under different co-culture conditions.

### 2.5 Statistical analysis

All experiments were conducted in triplicate biological replicates with at least three independent experiments. Statistical analysis and graphing were conducted using GraphPad Prism version 10.0 (GraphPad Software, San Diego, CA, USA). For comparisons between two groups, a paired two-tailed *t*-test was used. For comparisons involving more than two groups, one-way analysis of variance (ANOVA) followed by appropriate post hoc tests (e.g., Dunnett's multiple comparisons test, Holm-Šídák's multiple comparisons test) was performed. Differences were considered statistically significant when *P* < 0.05. Data are presented as mean ± standard deviation (SD).

## 3 Results and discussion

### 3.1 Artificial mucus performs the essential physical barrier function

In previous synthetic mucus formulations, lipid components such as linoleic acid, cholesterol, and phosphatidylcholine were included to mimic the amphiphilic nature of native mucus. These lipids influence mucus viscosity, hydrophobic barrier properties, and particle entrapment, especially under shear stress or when modeling intestinal motility. However, their inclusion requires emulsification agents like polysorbate 80, and prolonged exposure to these surfactant-lipid mixtures can damage epithelial viability in static co-culture setups.^17^ In this study, to enable prolonged co-culture for up to 48 hours rather than the short 1–2 hour overlay typically used in biocompatibility assays, we excluded lipids from the artificial mucus formulation.

**Figure 1.**
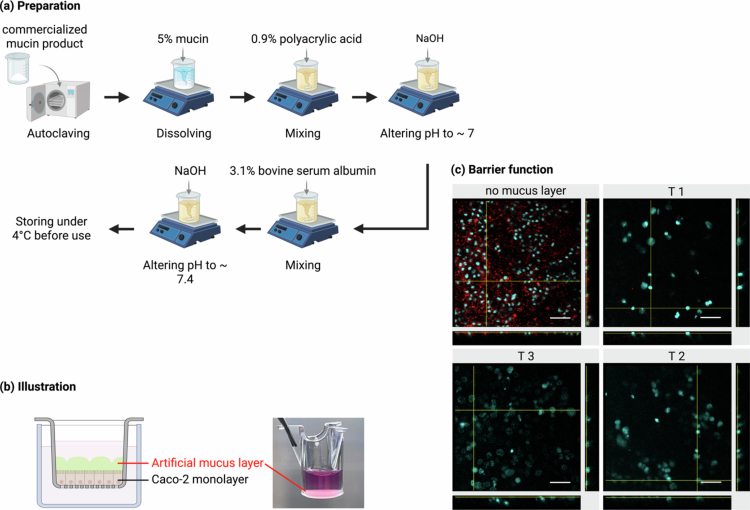
Construction and validation of an artificial mucus layer for *in vitro* co-culture. (a) Schematic of artificial mucus preparation: commercial porcine mucin was autoclaved, solubilized, and thoroughly mixed with polyacrylic acid (PAA) and bovine serum albumin (BSA). The pH was adjusted stepwise to ~7 and then 7.4. (b) Schematic illustration of the experimental setup showing the artificial mucus layer stably applied above a differentiated Caco-2 monolayer in a Transwell insert. (c) Confocal imaging of bacterial penetration after 24-hour incubation with 10^9^ CFU/mL of *L. plantarum* OLL2712. Caco-2 nuclei were stained blue with Hoechst 33342, while *L. plantarum* OLL2712 were labeled red with CellTracker Red CMTPX Dye. Three mucus layer volumes representing increasing thicknesses were examined: T1 (33 μL), T2 (66 μL), and T3 (99 μL). The no mucus layer condition was used as a control. Scale bars = 50 μm.

To construct a physiologically relevant mucosal barrier, we formulated artificial mucus composed of 5% porcine mucin, 0.9% PAA, and 3.1% BSA, adjusted to pH 7.4 with a final osmolality of about 300 mOsm/kg—closely matching intestinal conditions (Figure 1a). As shown in Figure 1b, the mucus layer formed a clearly distinguishable lower phase, stably separated from the cell culture medium above, with no visible mixing or floating. This distinct two-layer structure remained stable throughout the 48-hour culture period, demonstrating that the artificial mucus had sufficient structural integrity to stay intact under experimental conditions. This feature is crucial for maintaining consistent barrier function and spatial separation during subsequent dynamic host–microbe interaction studies.

To evaluate the barrier capability, Caco-2 monolayers (stained blue) were cultured on Transwell inserts and overlaid with artificial mucus of increasing thickness: T1 (33 μL), T2 (66 μL), and T3 (99 μL). A high concentration of live *L. plantarum* OLL2712 (stained red) was then added to the apical compartment to visualize bacterial distribution by confocal fluorescence microscopy clearly. As shown in Figure 1c, in the absence of a mucus barrier, bacteria were detected in direct contact with the epithelial cells, indicating direct bacterial exposure. In contrast, a thin mucus layer (T1) significantly reduced the bacterial proximity, while increasing the thickness to T2 and T3 effectively prevented bacterial penetration into the epithelial compartment. Orthogonal projections further confirmed that the bacterial signal was not detected either at the cell surface or within approximately 30 μm above the cell layer when the mucus layer was present, demonstrating the essential physical barrier function of the mucus layer to prevent cells from direct contact with bacteria.

These observations confirm that the artificial mucus effectively mimics the essential physical properties of the native intestinal mucus layer, shielding host cells from direct bacterial exposure. This is particularly important for supporting longer-term co-culture with live microbes, as it is typically the time-limiting factor in direct-contact systems due to cytotoxicity. By preventing epithelial damage while still allowing bacterial metabolite diffusion, the model provides a more physiologically relevant microenvironment for examining functional host responses. This marks a significant improvement over traditional systems that rely on non-viable bacteria or short-term exposure for *in vitro* microbiota research, probiotic screening, and inflammation modeling.

### 3.2 Artificial mucus demonstrates good biocompatibility with various thicknesses

**Figure 2.**
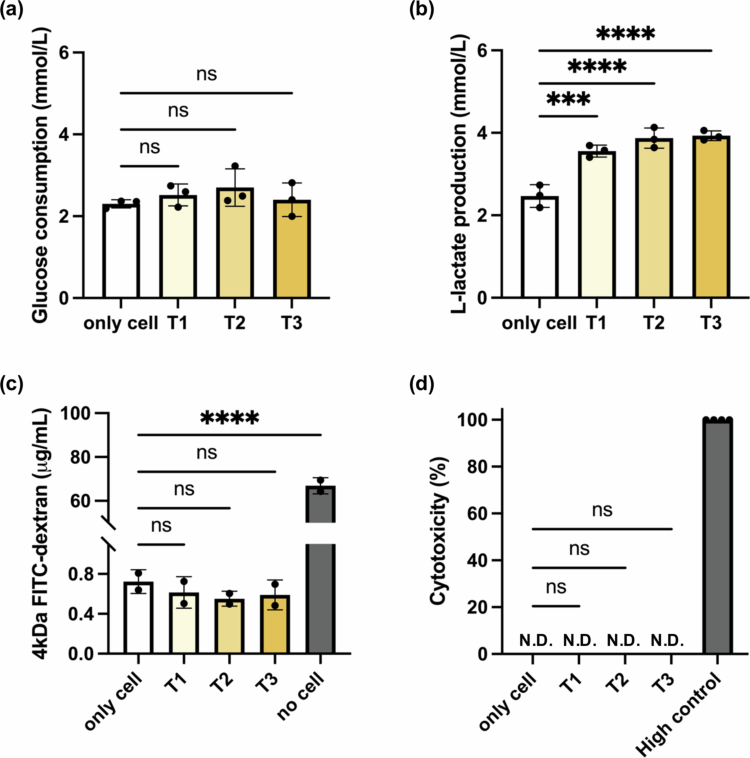
Assessment of epithelial viability and barrier integrity after 48-hour culture under varying mucus thickness. (a) Glucose consumption and (b) L-lactate production in the culture medium, reflecting cellular metabolic activity after 48 hours. (c) Epithelial barrier integrity assessed by 4 kDa FITC-dextran permeability assay. (d) Cytotoxicity evaluated by LDH release from Caco-2 cells into the basolateral medium. Data are presented as mean ± standard deviation (SD), *N* = 3. N.D.: not detected. Statistical significance was determined by an ordinary one-way ANOVA with appropriate post hoc tests, comparing each treatment group with the no-mucus control. ns: not significant; ***p < 0.001; ****p < 0.0001.

To assess the biocompatibility of the artificial mucus, varying volumes of the mixture (33 μL, 66 μL, and 99 μL) were applied onto 14 d differentiated Caco-2 monolayers, corresponding to different thicknesses of the artificial mucus layer (T1, T2, and T3, respectively). After 48 hours of incubation, metabolic activity, cell viability, and barrier integrity were evaluated under each condition (Figure 2).

As shown in Figure 2a, glucose consumption remained steady across all groups, indicating that the presence of the mucus layer did not negatively impact Caco-2 metabolism even at greater thicknesses. In contrast, L-lactate production (Figure 2b) increased significantly with thicker mucus layers. This trend probably reflects reduced oxygen availability caused by the diffusion-limiting nature of the mucus barrier, consistent with the hypoxic microenvironment observed in the intestinal lumen.^19^ These findings suggest that the artificial mucus construct not only maintains normal cellular metabolism but also contributes to the establishment of a physiologically relevant oxygen gradient.

Epithelial barrier integrity was further assessed using a 4 kDa FITC-dextran permeability assay (Figure 2(c)). All mucus-treated groups (T1–T3) exhibited similarly low levels of FITC-dextran diffusion, comparable to the control group with only cells, indicating that the presence of the artificial mucus layer did not compromise tight junction function or cause paracellular leakage. In contrast, the no-cell control exhibited significantly higher permeability, confirming assay specificity. Notably, the mucus layer itself did not block the diffusion of the 4 kDa molecule, as the permeation profile remained consistent across different mucus thicknesses. This aligns with previous findings that low-molecular-weight solutes can easily diffuse through mucin-based hydrogels and that mucus does not significantly hinder nutrient-sized molecules under physiological conditions.^20^

Cytotoxicity was also assessed using an LDH release assay (Figure 2d). All mucus-treated groups showed low and statistically similar LDH levels compared to the control with only cells, while the positive control group exhibited nearly complete cell lysis. These results confirm that the artificial mucus formulation and its application do not induce toxicity to the epithelial monolayer.

Taken together, these findings demonstrate that the artificial mucus layer, regardless of its thickness, is biocompatible with epithelial cells over an extended 48-hour culture period in this study. Furthermore, it enables the formation of an oxygen gradient and maintains epithelial barrier integrity, which are essential for creating a physiologically relevant host–microbe interface *in vitro*.

### 3.3 Artificial mucus supports metabolic activity and proliferation of *Lactiplantibacillus plantarum* OLL2712

Following validation of barrier function and biocompatibility, *L. plantarum* OLL2712 were cultured for 48 hours in antibiotic-free cell culture medium, with or without the artificial mucus layer, to evaluate the ability of the mucus system to support bacterial viability and metabolic activity.

*L. plantarum* OLL2712 is a proprietary probiotic strain developed by Meiji Co., Ltd., known for its potent anti-inflammatory and immunomodulatory effects.^21^ Previous studies in both animal models and human trials have demonstrated its beneficial roles in maintaining gut barrier integrity, modulating immune responses, and improving metabolic health.^22-25^ These properties make *L. plantarum* OLL2712 a suitable candidate for studying probiotic-host interactions under physiologically relevant conditions.

**Figure 3.**
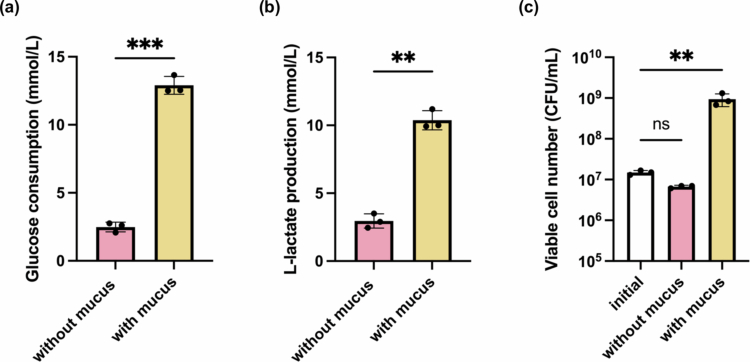
Assessment of bacterial metabolic activity and proliferation in the presence or absence of artificial mucus. (a) Glucose consumption (b) L-lactate production as markers of bacterial metabolic activity by *L. plantarum* OLL2712 after 48 hours of culture. (c) Endpoint viable bacterial cell number count (CFU/mL) compared to the initial inoculum. Data are presented as mean ± standard deviation (SD), *N* = 3. Statistical analysis was performed using a paired two-tailed t-test or an ordinary one-way ANOVA with appropriate post hoc tests. ns: not significant; **p < 0.01; ***p < 0.001.

To assess the strain's metabolic activity, glucose consumption and L-lactate production were quantified after 48 hours, with and without the presence of the artificial mucus layer. As shown in Figure 3a, glucose consumption in the mucus-containing group was significantly higher than in the bacterium-only control, indicating enhanced microbial carbohydrate utilization. Consistently, Figure 3b showed increased L-lactate production under the mucus-containing group, reflecting elevated fermentative metabolism. These findings confirm that artificial mucus matrix supports sustained bacterial metabolic activity.

Furthermore, viable CFU counts (Figure 3c) revealed a robust approximately 100-fold increase only when the artificial mucus layer was present. Without mucus, viable CFU counts remained similar to initial levels, indicating minimal growth. This highlights the crucial role of the artificial mucus layer in establishing a suitable microenvironment for colonization and growth.^8^^,^^18^

**Figure 4.**
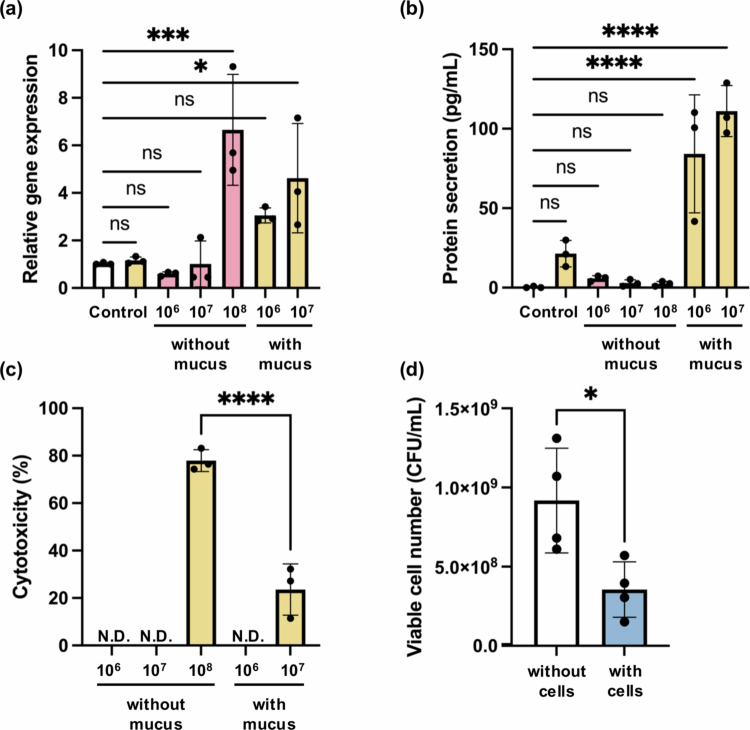
Assessment of the artificial mucus layer in recapitulating host-microbe interaction. (a) Relative gene expression of hBD-2 in Caco-2 cells after 48-hour co-culture with *L. plantarum* OLL2712 at increasing bacterial densities (10⁶–10⁸ CFU/mL) with or without the artificial mucus layer. (b) hBD-2 protein secretion measured by ELISA under corresponding conditions. (c) LDH release from Caco-2 cells indicating epithelial cytotoxicity. (d) Viable bacterial counts in mucus-only vs. mucus with epithelial cells, assessed after 48-hour co-culture. Control includes cell-without-mucus (white bar) and cell-with-mucus (yellow bar) groups. Data are presented as mean ± standard deviation (SD), *N* = 3–4. N.D.: not detected. Statistical analysis was performed using a paired two-tailed t-test or an ordinary one-way ANOVA with appropriate post hoc tests. ns: not significant; *p < 0.05; ***p < 0.001; ****p < 0.0001.

Although mucins can serve as carbon sources under nutrient-limited conditions, the artificial mucus layer in this system is unlikely to act as the primary nutrient source for *L. plantarum*. This model was maintained in DMEM containing a high concentration of glucose as a readily available substrate, and significantly increased glucose consumption was observed in mucus-containing conditions (Figure 3a), indicating that glucose remained the dominant metabolic input. Moreover, epithelial function and cell viability were preserved throughout the co-culture period (Figure 4), suggesting that extensive mucus degradation did not occur. Therefore, rather than mucus serving as a direct nutrient substrate, we interpret the enhanced bacterial activity in the artificial mucus system as primarily attributed to microenvironmental regulation.

First, the hydrogel matrix reduces diffusion rates of nutrients, allowing substrates like glucose to accumulate locally near bacterial colonies. This nutrient-retentive effect enhances bacterial metabolic efficiency by maintaining a more stable supply of carbon sources.^26^ Second, the mucus provides a physical structure for bacterial aggregation and adhesion, thereby mimicking *in vivo* biofilm-like organization and increasing local cell density.^27^ Third, the mucus may buffer against oxidative stress and rapid pH changes, which could otherwise damage bacterial viability when they are directly exposed to the culture medium. This protective role aligns with the *in vivo* function of mucus as a physicochemical shield for resident microbiota.^28^

Importantly, these results emphasize that bacterial viability is not solely affected by nutrient supply, but is also profoundly influenced by spatial and physicochemical context. The artificial mucus in this study effectively recreates such an environment, supporting both host compatibility and microbial proliferation. This feature is essential for modeling dynamic, physiologically relevant host–microbe interactions over extended periods.

### 3.4 Artificial mucus involved *in vitro* model enables a dynamic host–microbe interaction loop of hBD-2 response induced by probiotic *Lactiplantibacillus plantarum* OLL2712

To assess the ability of the artificial mucus layer in recapitulating host–microbe interactions, we chose human β-defensin-2 (hBD-2) as a representative epithelial response marker in response to probiotic stimulation. hBD-2 is an inducible antimicrobial peptide secreted by intestinal epithelial cells in response to microbial stimulation, primarily through Toll-like receptor (TLR)-mediated signaling cascades.^29^ Previous research also demonstrated hBD-2 induction via NF-κB and AP-1 binding sites.^30^ Functionally, hBD-2 contributes to mucosal defense not only by directly suppressing microbial overgrowth but also by enhancing the epithelial barrier. Its upregulation is a commonly reported immunomodulatory effect among probiotic strains, making it a reliable indicator of epithelial activation in co-culture systems. Building upon previous work showing that *L. plantarum* OLL2712 demonstrates beneficial roles in host health regulation,^16^^,^^21^^,^^22^^,^^25^ this study extends its application to a mucus-integrated co-culture model to examine dynamic host–microbe interactions.

Caco-2 cells were co-cultured with *L. plantarum* OLL2712 at different initial densities (10⁶, 10⁷, 10⁸ CFU/mL) for 48 hours, either with or without the mucus layer in between. As discussed earlier for Figure 3c, bacterial proliferation was significantly affected by the presence of mucus. Without mucus, bacterial counts mainly remained constant, indicating limited growth and a constant exposure ratio of bacteria to epithelial cells. In contrast, mucus-supported cultures reached approximately 10⁹ CFU/mL, showing robust proliferation. As a result, even with the same starting inoculum, bacterial exposure dynamics varied substantially depending on the presence of mucus, leading to different magnitudes of epithelial stimulation ratio over time. This distinction is critical for interpreting downstream host responses, as cells cultured with mucus likely encountered higher levels of bacterial effects, as they were subjected to dynamic, increasing microbial exposure over time.

Across all conditions, the RT-qPCR results (Figure 4a) revealed that hBD-2 gene expression increased in a dose-dependent manner, aligning with previous findings on probiotic-induced defensin expression.^30^ Importantly, in the presence of mucus, significant upregulation was observed even at lower bacterial doses, whereas in the absence of mucus, only the highest dose (10⁸ CFU/mL) induced notable gene expression. This suggests that the mucus environment facilitates more efficient microbial sensing by the epithelium, potentially by enhancing microbial proximity while mitigating cellular stress.

However, ELISA quantification of secreted hBD-2 protein (Figure 4b) revealed a striking dependence on mucus layer: hBD-2 secretion was only detectable in with-mucus groups (84.2 ± 37.1 pg/mL at 10⁶ CFU/mL and 111.1 ± 16.0 pg/mL at 10⁷ CFU/mL in 24-well insert). In contrast, no secretion was observed in the absence of mucus, despite elevated transcription under high-dose conditions. Paolillo et al. similarly added viable *Lactobacillus plantarum* directly onto Caco-2 monolayers cultured in six-well plates and detected 32 ± 2.4 pg/mL of hBD-2 after 48 hours per well.^31^ Notably, hBD-2 secretion levels in our model were approximately 100-fold higher than those reported previously, when normalized to epithelial surface area under comparable bacterial exposure ratios, highlighting the enhanced physiological relevance of the mucus-supported co-culture system.

LDH cytotoxicity analysis (Figure 4c) confirmed that without mucus, Caco-2 cells exposed to 10⁸ CFU/mL bacteria experienced high cytotoxicity of about 80%, which likely impaired protein translation and secretion capacity. In contrast, the mucus layer preserved cell viability (≤20% cytotoxicity), allowing for the complete execution of the immune response, from transcription to protein secretion. This highlights the dual role of mucus in spatial shielding and functional preservation of epithelial responses.

It is worth noting that endpoint CFU counts (Figure 4d) were significantly reduced in mucus systems containing epithelial cells compared to cell-free controls. This suggests an emergence of a negative feedback loop, potentially mediated by secreted antimicrobial factors such as hBD-2, which are known to limit the overgrowth of Gram-positive bacteria.^32^^,^^33^ Alternatively, nutrient competition between host and bacteria could also contribute to growth suppression, similar to that under *in vivo* conditions.^34^

Also, in preliminary co-culture tests with other non-probiotic strains,^30^ such as *Escherichia coli* (NBRC 3301), the barrier integrity of the Caco-2 monolayer was gradually compromised, as indicated by a rapid decline in trans-epithelial electrical resistance (TEER) within 12 hours of co-culture and the bacterial penetration into the basolateral compartment. This behavior likely reflects the higher motility and proliferation rate of *E. coli*, the non-probiotic properties, and the absence of antimicrobial or immune components in the current setup. Therefore, we focused on *L. plantarum* as a probiotic model to evaluate the supportive role of the artificial mucus layer in maintaining barrier integrity and modulating the host response. Future adaptation of the system may enable the integration of immune cells or antimicrobial factors to investigate interactions with pathogenic strains.

In summary, our model recapitulates a dynamic and physiologically relevant host–microbe interaction loop: (i) microbial presence induces epithelial hBD-2 transcription, (ii) mucus preserves cell viability for effective hBD-2 secretion, and (iii) the secreted peptides subsequently restrict further bacterial overgrowth. This self-contained feedback loop captures essential features of mucosal immunity and microbial regulation. The artificial mucus-intergrated model thus provides a robust, scalable, and animal-free platform for dynamic host–microbe studies, with strong translational potential in probiotic screening and intestinal disease modeling.

## 4 Conclusion

In this study, we present an *in vitro* intestinal model incorporating a structurally stable artificial mucus layer that enables 48-hour live interaction between *L. plantarum* OLL2712 and human intestinal epithelial cells. Unlike conventional systems that have a limited mucus barrier or rely on non-viable bacteria, our physiologically relevant co-culture model supports sustained microbial growth while preserving epithelial viability and barrier function simultaneously. Most notably, this model successfully recapitulates a complete dynamic host-microbe interaction loop, comprising bacterial stimulation, epithelial secretion, and microbial suppression. This dynamic loop mirrors the *in vivo* host response and highlights the critical role of the mucus layer in modulating epithelial immune function.^35-37^

Beyond demonstrating biological relevance, this model offers several key advantages over animal-based approaches, including real-time sampling, controlled microenvironments, and compatibility with a diverse range of microbial strains. Given that mucus layer disruption is a hallmark of IBD, this system provides a tractable and scalable platform to investigate how specific microbes influence epithelial homeostasis and mucosal defense. This platform bridges the gap between simplified *in vitro* assays and complex *in vivo* systems, holding broad potential for applications in probiotic development, microbiota-driven disease modeling, and personalized research on gut health. Its scalability, reproducibility, and compatibility with conventional culture formats make it a promising platform for both academic and industrial applications.

## Disclosure of potential conflicts of interest

Y.W. and T.S. are employees of Meiji Holdings Co., Ltd. All other authors declare no potential conflicts of interest.

## Data Availability

The authors confirm that data supporting the findings of this study are available within the article or upon request from the corresponding author.
